# Qualitative Analysis of Feedback From a Primary Care Resident Clinic

**DOI:** 10.7759/cureus.109213

**Published:** 2026-05-19

**Authors:** Shelley R Ost

**Affiliations:** 1 General Internal Medicine, University of Tennessee Health Science Center College of Medicine, Memphis, USA

**Keywords:** continuity clinic, feedback, graduate medical education, primary care, residency, teaching clinic

## Abstract

Background

Residents spend a great deal of time with supervising faculty in their continuity clinic in primary care residencies. The long-term relationship between the resident and faculty provides an opportunity to provide feedback on the resident’s knowledge, skills, and practice style.

Materials and methods

Focus group discussions with primary care residency supervising faculty at the University of Tennessee Health Science Center were analyzed to determine the goals, current knowledge, feelings, and barriers regarding providing feedback to residents during continuity clinic. Qualitative analysis using grounded theory identified themes in the focus group data, leading to an underlying theory of feedback provision to residents in a primary care resident clinic. Data were used to propose improvements to the current feedback process.

Results

Categories in the data included the longitudinal nature of the faculty-resident relationship, the need to manage emotional responses to feedback, faculty preparedness, and the difficulty of determining the content of the feedback to be provided. These influenced the three significant components of feedback: content, delivery, and reception.

Conclusions

Addressing components of feedback content, delivery, and the learner's attitude or receptivity can improve the quality and quantity of feedback given to residents in the clinic.

## Introduction

Residency education in clinical settings is based on an apprenticeship model, with attending physicians modeling or observing clinical care. To learn and improve, residents need feedback on their performance during clinical rotations. Though formal evaluation of residents on rotations is a requirement from the Accreditation Council for Graduate Medical Education (ACGME) [1], and various formats and suggestions for giving feedback have been proposed [2-4], many higher education institutions, residency programs, and medical schools struggle with providing meaningful feedback to medical learners [3,5-7].

Research hypotheses

Factors anticipated to influence faculty feedback to residents, based on the investigator's previous experience and a review of the literature, include faculty members’ comfort level with providing feedback, prior education on how to give feedback, time constraints, and managing the emotional context. For both the faculty member and the receiving resident, these factors are key to the provision of feedback, including its timing and quality.

The length and depth of the longitudinal relationship between the supervising continuity clinic faculty member and resident (including the frequency of opportunities for observation by faculty) serve as key factors in facilitating the provision of quality feedback.

Furthermore, it was hypothesized that the formal evaluation process was poorly aligned with the provision of feedback on timing, content, and delivery.

Significance of the Problem

The ACGME recognizes that faculty members’ formative feedback to residents is a crucial part of residents' learning and encourages the timely provision of such feedback [8]. At the author's institution, learners in our medicine and pediatrics residency program completed the 2019 ACGME survey, reporting dissatisfaction with feedback received after rotations, indicating that our program, like many, struggles to provide residents with formative feedback. Only 71% of our residents were satisfied with feedback after rotations, which was below the national average of 73% and lower than satisfaction with most other aspects of the program. In a conversation with the primary author, one resident described working in a clinic with his attending physician as “four years of radio silence.” Our program has recognized this as a critical need, given the importance of a culture of formative feedback in promoting improvement in the apprenticeship model [9].

Assumptions and Limitations

One limitation of this study is that the limited number of participating faculty did not ensure that the focus groups achieved redundancy in content. In addition, using a single institution, despite three different clinic settings among the faculty members, limits the generalizability of the findings. However, using our own institution for data collection provides much more specific data for an institutional quality improvement process. The potential limitation of faculty members' reluctance to share personal opinions with colleagues was addressed by allowing anonymous email comments after the focus group. A final limitation is that only faculty members participated in the focus groups, and no residents. This was due to concerns from the Institutional Review Board about the authority relationship between the primary author (who was previously an associate program director in one of the programs) and the residents who would have participated in the focus groups. Having the perspective of the residents seeking and receiving feedback would have allowed a more broadly focused study that would have provided perspectives from both sides of the feedback dyad.

Review of related literature

Feedback, as it relates to medical education, has recently been defined as a two-way conversation in which preceptors provide an assessment of a learner’s skills and behaviors, a comparison with expected or ideal levels, and a plan to close the gap. There is a well-recognized component of learner participation in internalizing feedback content and using it to change behavior and engage in additional learning activities [10,11].

Studies in various disciplines have investigated the usefulness and mechanics of individual performance evaluations. Summarizing these findings, a review of the literature in medicine and industry confirmed that, in industrial settings, performance evaluations can be associated with poor performance and loss of self-confidence, and that the perception of feedback by the person being evaluated is critical to acceptance and growth [6]. This study found similar themes in industry as in medical education. A qualitative study of medical students, athletes, and musicians found very similar desires for feedback and factors affecting acceptance of feedback across the three fields of study [12]. Factors included the learning culture, the learner's individual characteristics, perceptions of the feedback's motivational impact and specificity, and the learner’s receptivity. An extensive literature review found that characteristics of feedback to physicians most likely to change behavior included the source of the feedback and the duration of the follow-up cycle [13].

More specifically, regarding residency education, Ramani et al. examined the learning culture in relation to face theory. They proposed that saving “face,” or self-concept, was a contributing factor in both learners’ willingness to accept feedback and supervisors’ reluctance to give constructive criticism [14]. Tailoring feedback to the learner was explored in a study of preceptors providing feedback on a videotaped discussion of goals of care, including cases involving an emotionally distressed resident, a resident who did not accept feedback, and a high-performing resident [15]. This study found that asking reflective questions was often ineffective at guiding residents to identify areas for improvement. Faculty members used a variety of approaches, including coaching, mediating, mentoring, and directing resident behavior. Acknowledging and working through emotional responses to feedback helped learners accept constructive feedback and develop an action plan in a study of general practitioner training [2]. For the most part, these studies did not identify the types of feedback that learners would find helpful in changing behavior. Instead, they focused on the giving and acceptance of feedback and on barriers to giving feedback in general and to giving constructive criticism in particular.

Residents in primary care specialties perform much of their daily patient care and learning activities under the direct or indirect supervision of attending physicians. Experienced supervisors can significantly influence their residents' learning across multiple areas, such as communication, physical examination skills, and diagnostic reasoning, by providing timely feedback and identifying learning opportunities during routine clinical rotations. Medical learners desire specific feedback on how to improve [2]. Studies have found that promoting a growth culture and modifying the delivery of feedback can improve its acceptance by learners [8]. Our study explored the type of feedback given to primary care residents in an outpatient clinical setting and barriers to faculty providing that type of feedback. Findings will be used to improve the feedback process at our institution and address deficiencies identified in our ACGME resident survey. A multimodal approach will be needed to improve the process, as faculty delivery of feedback and the learning culture affect feedback acceptance, emotional response to feedback, and motivation [6].

At other institutions, improvements in faculty feedback provision have been documented following targeted interventions. In one study, using feedback reminder cards based on the ACGME competencies and dedicated feedback time improved both the quality of feedback given to residents on an inpatient rotation, including increasing the amount of constructive feedback given, and also improved faculty members’ comfort with feedback and residents’ perception of the amount of feedback given [16]. A similar study among medical students on a psychiatry rotation used a smartphone app and found that it was generally acceptable, helped guide the feedback process, and provided a useful reference by recording a written summary of the verbal feedback. Users benefited from pre-populated “scaffold” feedback that guided faculty in addressing commonly seen performance gaps, though learners did not find it as valuable. The smartphone app used to complete workplace-based assessments was found to be somewhat distracting and to depend on the operator's technical knowledge [17]. A faculty development session using a “flipped classroom” approach showed overall improvement in the quantity, quality, and timeliness of feedback, as measured by faculty pre- and post-course surveys [8].

One promising model of feedback explored in a few studies was the R2C2 feedback model, which stands for relationship building, reaction to feedback, content understanding, and making a plan for change [18]. In this model, steps include asking the learner for a self-assessment, providing feedback, addressing the emotional response and understanding of the content, and making a team-improvement plan to work toward it using the relationship with the supervisor. Further studies using this model have shown that it promotes mutually goal-oriented, collaborative discussions between faculty and residents to guide residents’ future growth. However, there is concern that it may be less applicable to struggling residents [19]. Milan, Parrish, and Reichgott described using either the PEARLS (Partnership, Empathy, Apology, Respect, Legitimation, Support) framework or the transtheoretical “stages of behavior change” model to address faculty feedback, particularly noting that the transtheoretical model helped address serious deficiencies in learner performance [20]. The concept of an “educational alliance” between learner and teacher has been recognized by some, along with the importance of the learner’s reception of teacher feedback [11].

The value of feedback in medical education is widely recognized. In a study of emergency medicine residents, receiving feedback increased the likelihood of reporting near-misses, which was attributed to the promotion of a growth culture and to increased learners’ ability to recognize near-misses and bring them to faculty members [21]. Among medical students and residents in a Canadian study, 95.6% said that giving constructive feedback was an important role for a preceptor, and 90% desired timely feedback [22]. Feedback was found to influence learners' behavior when accepted by the receiving learner, especially when the supervisor was known and respected and had a longitudinal relationship with the learner [23]. Finally, feedback on performance from supervising physicians to residents constitutes the bulk of the learning activities in a residency program, dwarfed by standardized testing and didactics in terms of time commitment and volume of information given.

## Materials and methods

Population

Faculty from primary care residency programs at the University of Tennessee Health Science Center (UTHSC), including internal medicine, internal medicine/pediatrics, pediatrics, and family practice, constituted the study population. In these residency programs, residents may see patients in the continuity clinic weekly or monthly and are usually assigned to one to three attending physicians who follow them throughout their three- to four-year residency. Faculty participating in the focus groups varied in experience from one to 18 years of supervising residents in a primary care clinic at the UTHSC.

Setting

Outpatient faculty from the primary care specialties at UTHSC comprised the faculty focus groups. These faculty members supervise the same group of residents who provide primary care to a panel of patients for two to four years of residency. They may provide longitudinal assessments of the residents’ performance, including knowledge gaps and areas for improvement. Some studies have shown that learners accept feedback better in the context of a longitudinal relationship with a faculty member [5,7,14]. Outpatient faculty members also have the advantage of following their learners’ performance over time to see if they have enacted behavior change or improved knowledge or skills based on prior feedback. Because the faculty members in this study are all from primary care-focused residencies, they have a common set of skills they are trying to develop that do not involve as many technical skills as performing surgery.

Email invitations were sent to faculty members in the UTHSC College of Medicine, including the internal medicine, pediatrics, and family medicine residency programs. No response was received from the family medicine program, so its faculty did not participate.

Study design

Two faculty focus groups, one of four and one of six members, were arranged from among those who responded to an email invitation, including faculty members who supervise residents on outpatient primary care rotations. A total of ten faculty members participated out of the 20 approached. They were divided into groups based on their interview availability. Both groups were mixed among the internal medicine, medicine, and pediatrics faculty. Focus groups were held virtually, via Zoom (Zoom Communications, Inc., San Jose, CA, USA), and moderated by the primary author, who has experience in qualitative research. See Appendix 1 for focus group questions. Later, the primary author sent an anonymous Google Forms (Google LLC, Mountain View, CA, USA) survey to all subjects, asking them to share anything they did not feel comfortable saying to the group or in front of the moderator (see Appendix 2). The UTHSC College of Medicine Institutional Review Board approved this project (approval number: 21-07969-XM).

Data analysis

Data were analyzed using constructivist grounded theory to identify themes from transcripts of the focus group meetings. Constructivist grounded theory allows for interpreting the data as it is obtained, without a preconceived notion of results. It also takes into account the underlying or interpreted meaning behind statements and actions rather than only the statements or actions themselves [24]. The focus group discussions were audio-recorded and hand-transcribed by the primary author. The focus group questions and discussion prompts were refined as data collection proceeded.

Dr. Ost coded the transcripts and developed a codebook, which was shared with secondary coders Dr. Smith and Dr. Anna Quantrille Allen. Any differences were resolved by discussion, and only codes agreed upon by all were used. Codes were categorized, with direct quotes compiled into a shared file. Themes were identified through the review and comparison of transcripts, using the constant comparative method. Both expected and unexpected themes were considered and recorded during analysis. An underlying theory of the feedback process was developed, comparing and contrasting it with prior studies in other medical education research. The theory was then used to propose changes to the evaluation and feedback process to increase the quantity, quality, and reception of feedback from faculty members to residents in the primary care residency continuity clinic.

To establish data validity, three researchers with experience in medical education reviewed the coded data and the codebook. Additionally, the data and the underlying theory were triangulated with prior studies on the same topic, as well as with similar studies from residency programs and other medical education settings. Other factors affecting validity included careful subject selection to best represent the population under study and an iterative data-collection process, with questions and prompts refined between focus groups. Validity was also established by the presence of similar concepts between the three different focus groups.

## Results

Factors that make up feedback in primary care residency fell into three themes: the content of the feedback to be provided, the delivery, and the learner's receptivity. Sub-categories of feedback content included the specificity and objectivity of feedback, whether it was medical knowledge or non-medical, global vs. incident- or patient-based in scope, and positive/reinforcing vs. negative/constructive. Other ideas mentioned in the focus groups that influenced multiple themes included group evaluations and the perceived value of faculty feedback to residents.

Feedback content that faculty desired to provide included highly specific, direct-observation-based content, both positive/reinforcing and negative/constructive, regarding both medical and non-medical skills (Table 1). In fact, most of the discussion in all three focus groups centered on how to develop the feedback content and tailor it best to the learner and the learning environment. The first dichotomy presented was the difference between formal and informal feedback. The groups defined formal feedback as a separate, private, sit-down conversation in which global feedback was given to the resident. This formal feedback was often, but not always, related to filling out an official evaluation. According to the focus groups, informal feedback occurred regularly in smaller doses, termed “micro-feedback” by one focus group member. It was based on a particular patient or event, and the goal was to provide this type of feedback on most encounters or at least at each clinic session. Faculty felt that this patient- or event-focused “micro-feedback” was the bulk of feedback given to residents during their continuity clinic observation. Feedback was felt to be easier to give when a resident's performance was remarkable or poor; identifying appropriate feedback content was much more difficult for faculty members when a resident was performing well but not remarkably so.

**Table 1:** Considerations for content of feedback provided to residents

Content	Description	Example
Positive vs. negative	More comfort giving positive	I try to give individual feedback, mainly just positive feedback, when I think a resident has a complicated patient and I think they’ve done a really good job… I’m better at giving positive feedback than negative feedback.
Medical vs. non-medical	Medical knowledge or patient management skills feedback vs. communication skills, patient counseling, workflow	You don’t know the cholesterol numbers, and in order to manage cholesterol, you have to know that, so when we go back in the room, let’s open up the chart and look that up, and you didn’t know what the vitamin D level was and they have osteoporosis, so you need to know that. I think that the difference between like a good resident and a great resident has to do with anticipation, customer service, and time management. But getting used to your flow is just as, if not more important than the medical knowledge at times.
Easier with outliers	Difficulty identifying content or how to instruct residents to improve, especially if performance was fairly good	If I’m thinking they’re doing a particularly good or bad job, I’ll advise them, or if I feel like they’re doing a particularly lukewarm job, but I know they have the ability to do better. I was just going to say, I don’t know if y’all just send me the residents that are really good, which I’m glad you do, but I don’t have to give much negative feedback to your residents. But for the majority that are just doing what they need to do and they’re very good, I kind of don’t deliver, because it’s not on my priority scale A, B, C, D, or E.
Specific	Learner-specific as well as incident-specific	Or not, “you need to be more attentive to detail.” You need to maybe provide some specific examples. The more specific, the better. In the discussion of the patient... you ask a question and they say "well, oh, I didn't think about that." In a lot of ways I think about that as a way I give specific feedback on individual patients.
Objective	Based on direct observation, about behavior or other objective observations rather than personal attributes or perceived source of behavior	I think the most important step is getting as much firsthand observation as possible. I try my best to avoid giving any feedback that I can’t give a firsthand account of... Because I feel like when it’s objective, there’s really no argument about it. “Hey, I notice that your note at this visit looks exactly similar to the one at previous visits,” not “Oh you just copy your notes all the time,” or “You must don’t like clinic because all your notes are copied.” Not drawing conclusions about their behavior, about what it means.
Negative/constructive	How to phrase or deliver negative feedback and provide direct next steps for learning or improvement	So phrasing it in a way that is a positive way, I guess. State a negative thing in a positive way is the way I usually try to do it. Then try to come up with some kind of action plan. And then have some kind of measurable way that the person could make improvement.

The specificity of feedback content was enhanced by the attending physician's direct observation, not only by being present in the clinic but also by observing the resident interacting with the patient. Focus group members recognized the value of feedback based on direct observation and, conversely, the pitfalls of providing feedback on events or interactions not witnessed firsthand. Next, faculty members emphasized that feedback should focus on objective data rather than the perceived root of the problem. For example, faculty members would point out that notes were copied and pasted, rather than attributing the observation to laziness or dislike of the clinic. “Diagnose the problem, not the learner” was a phrase that summed up this type of assessment.

Specifically tailoring feedback content to the learner was another major goal of the faculty members. They recognized the need to provide a different level of instruction to a first-year resident than to a resident nearing graduation. The topics discussed and the way they were presented, for example, by giving direct instruction on what to do rather than exploring the resident’s opinion or presenting multiple options, were used to appropriately tailor feedback to the learner's level of training and skill.

There were multiple comments from focus group members about how they found it difficult to give negative or constructive feedback, but easier to give positive feedback, reinforcing current behavior. Regardless of the faculty member's experience level, all felt less comfortable providing negative feedback. As discussed below, they recognized the importance of framing negative feedback so the learner is more receptive and better able to accept it; this leads to the next component: the delivery of feedback.

Delivery of feedback encompasses the setting, wording, and emphasis, as well as the types of feedback given in the same session. Delivery was also proposed to have a significant effect on its expected effectiveness. Faculty frequently described making an effort to present both positive and negative feedback in a positive light. Faculty wanted to encourage a growth mindset. One descriptive quote that illustrates this concept is, “It should be, ‘You’re on a journey, and this is the information you need to progress.”

Finding an appropriate time and place was seen as a significant barrier to delivering feedback to residents. Considerations for ideal feedback-giving sessions included a private area that was not too formal in setting (“In the dark conference room, it kind of feels a little bit more like detention”), set-aside time specific for feedback with no other tasks due at that time, and a combination of written recording for memory and verbal feedback for ease of two-way conversation.

By far the most significant barrier to providing feedback to residents throughout the focus group discussion was time or being too busy; the next-largest barrier was difficulty with the content and finding a way to deliver it so the learner would receive the feedback well.

Many faculty members expressed discomfort giving feedback, with fear of hurting someone’s feelings (“I had a recent conversation with a resident where they misinterpreted it, actually, what we wrote...they took that to mean that we meant that they were not making a good plan”), fear of lack of receptivity, and fear of lack of insight on the part of the resident. Residents were at times perceived as uninterested in feedback, defensive, or otherwise unaccepting of it. Faculty members noted that some residents consistently seemed receptive and ready to learn, while others were routinely perceived as uninterested or poorly receptive to faculty feedback. The less receptive attitude reduced faculty members’ willingness to provide feedback.

One situation found to mitigate the barrier of faculty discomfort occurred when a resident made a significant error. Faculty generally felt able to address that type of error in the moment and felt the need to do so to prevent similar errors in the future. The difficulty in sparing the learner’s feelings was acknowledged in this conversation by one focus group member, who said, “Like when a resident missed a calcium of 14.5, and I don’t think I really asked them how they felt about that; that’s a huge miss, you need to call the patient now.” However, the critical need to address a significant error outweighed the risk of hurting the learner’s feelings in this type of event.

Another theme in the data characterized the parties' attitudes in the feedback conversation: the faculty member's discomfort in giving the feedback and the receiving resident's attitude. Attitudes that promoted ease in giving feedback included a relationship with the resident in which the resident’s style of accepting feedback was known (“Some people like more tough love, and others like…you have to speak in a bit softer terminology”), and the belief that our feedback has value and provides constructive ways to allow a learner to improve behavior and skills. Faculty attitudes regarding feedback were inconsistent, and at times, we identified ambivalence even within the same faculty member. It was recognized that “students are craving feedback”; however, at times, the supervisors felt their formal evaluations were less valuable because they did not progress in the way medical student evaluations are used in formal performance evaluations for residency applications. Furthermore, some faculty members expressed that they were overwhelmed by the number of learners expecting feedback and formal evaluations, and therefore put off the process indefinitely.

**Figure 1:**
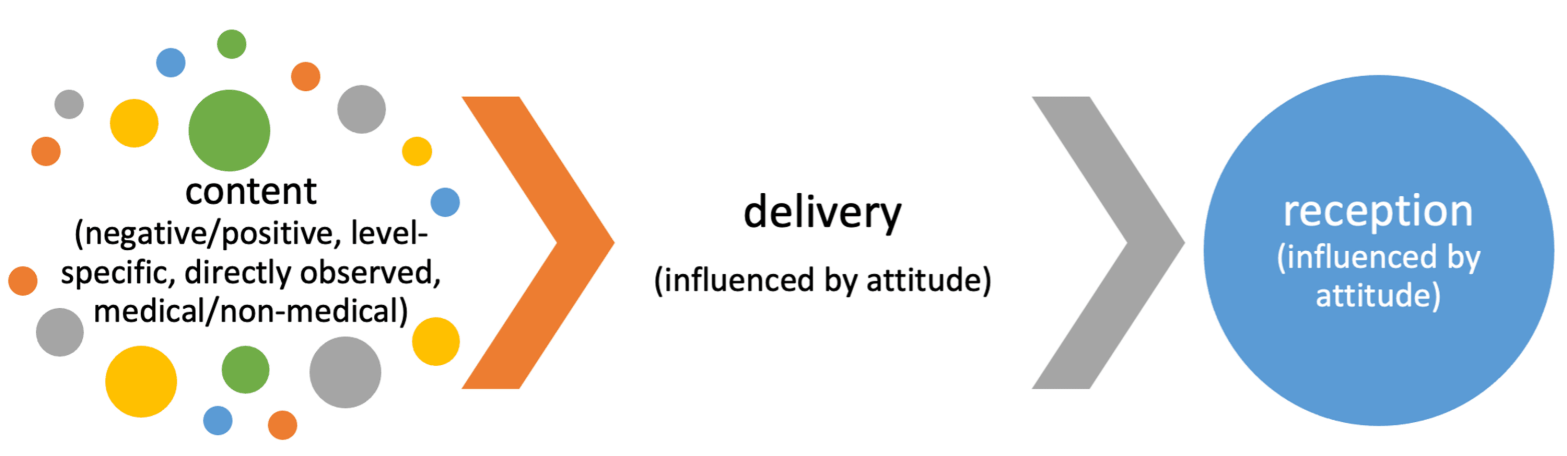
Theory of the process of resident feedback in the primary care clinic Image Credit: Dr. S. Ost

Significant interactions exist between content and delivery and between delivery and reception/attitude, in that, at times, faculty made an effort to include positive feedback alongside negative feedback to improve learners' emotional responses. As shown in the theory diagram below, drawn by the primary author, the content of the feedback significantly influenced how it was delivered, or, in some cases, whether it was given at all. Faculty members widely recognized the need to state things a certain way to improve reception. In turn, the learner's reception of feedback was directly affected by the mode of delivery, including the location, timing, and wording. Attitudes toward feedback significantly influenced faculty members’ willingness to give feedback and how they delivered it, to spare the learner's feelings. In sum, the data showed a process by which the content of feedback is conceptualized by the faculty member, delivered in a way influenced by the faculty member's attitude, and received by the learner in a way that is determined by the learner’s attitude toward both the content and the faculty member delivering it (Figure 1).

## Discussion

Focus groups revealed that faculty provision of feedback to residents in primary care continuity clinic was dependent on faculty members' ability to produce and feel comfortable addressing the content of feedback to be given; the delivery of feedback, both in terms of how to word it as well as the location, timing, and setting of feedback; and the learner’s reception of feedback. Addressing the three domains of content, delivery, and attitudes is predicted to improve feedback to residents in our program. As mentioned before, improving feedback to our continuity clinic residents is a goal of our residency programs, as reflected in our residents' verbal reports and in the ACGME survey.

The content of feedback, a major concern for faculty members in this study, can be enriched in a variety of ways. Faculty in this study emphasized the importance of direct observation of learners to provide specific examples, so opportunities for direct observation should be encouraged. Faculty also felt that suggestions for improvement were critical to learners' growth. Prior studies have shown improvements when pre-populated suggestions for feedback are combined with free-text comments. Providing suggestions can help spark memories of the learner’s observed performance and lead to more specific comments made in the learner’s evaluation. Reminders of basic concepts that were commonly agreed upon as important to feedback will also help guide the discussion; for example, reminding faculty that feedback should be timely, specific, objective, and behavior-focused, and that it should respond to directly observed behaviors or skills. Naming both medical knowledge and non-medical skills to evaluate, such as communication skills, differential diagnosis, and follow-up of test results, also prompts evaluators to remember specific events. Providing these reminder keywords on the form, app, or website used would help faculty conceptualize the feedback they should give.

Having an appropriate time and place to provide feedback was another significant factor. A deliberate but brief review process at the end of each ambulatory week, using a written, filed form for each resident with the above-mentioned reminders, would consolidate feedback from previous weeks for delivery at the quarterly evaluation sessions. This could be arranged as a group effort in a binder or file to be shared by all clinic attendings. Seeing others’ comments might remind faculty members of events they witnessed for a particular resident and reinforce areas that need improvement. This type of group evaluation would allow feedback from all supervisors to be combined, downweight outlying impressions, and identify trends. It would facilitate delivery by providing the content and setting a specific time for the discussion with the resident.

Despite years of experience, faculty members in the focus group expressed discomfort, particularly with providing constructive criticism. They recognized that a growth mindset is crucial to improving residents at all levels, but they did not feel they were “good at” giving negative feedback. Two skills that faculty used to increase their comfort with providing negative feedback included pairing it with positive feedback and phrasing it as a positive or as an area for improvement. The structure of faculty feedback included steps such as asking the learner to self-assess, addressing emotional responses, and developing an improvement plan, which closely follows the R2C2 model mentioned above. Using practice sessions with role-playing and instruction in managing negative emotions, along with growth-promoting instruction for learners, would help improve faculty members' comfort level and skill in delivering feedback. Such practice sessions, arranged in groups so faculty members can learn from each other, ideally, groups with faculty members with varying amounts of experience, would be a valuable faculty development activity. These sessions could be arranged specifically with primary care clinic faculty, since the longitudinal relationship with one’s clinic residents differs from many other resident-faculty relationships, which are rotation-specific and therefore shorter in duration. Addressing faculty members' delivery of feedback can potentially improve the learner's reception of feedback, though documenting this effect is outside the scope of this study.

Using the information gathered in this study, faculty can help address the three aspects of giving feedback to residents: content, delivery, and reception. Interventions to increase the availability of content, improve comfort with providing feedback, and manage learners' emotional responses are identified as targets to improve feedback to residents in primary care clinics.

## Conclusions

Interventions such as using faculty groups to discuss and combine feedback, teaching a feedback-provision process, and using reminders to stimulate recall of specific instances and skills for assessment are expected to improve faculty feedback to residents in a primary care clinic. These interventions would influence faculty members’ comfort level, the quantity of feedback given, the faculty members’ skill in delivering feedback, and, due to improved skill in delivery, would be anticipated to increase acceptance of feedback by the residents. In this way, the intervention program would address all of the major components of the feedback process as identified in this study.

## Appendices

Appendix 1: focus group questions

1. Do you provide individual feedback to residents periodically?

a. How often?

b. When?

2. Tell me how you provide feedback to residents.

3. What do you know about providing quality feedback?

a. Do you use this in your daily practice?

4. What are the barriers that you encounter in giving feedback?

5. What do you think of the evaluation process?

a. What would make it work better?

Appendix 2: final email survey question

Is there anything you can think of regarding feedback for residents that you did not think of during your focus group, or that you were not comfortable saying in a group setting?
